# Supplementary material for: Rapid degradation of histone deacetylase 1 (HDAC1) reveals essential roles in both gene repression and active transcription
Source: Nucleic Acids Res. 2024 Dec 19;53(4):gkae1223. doi: 10.1093/nar/gkae1223 (PMC11879047; doi:10.1093/nar/gkae1223)
Supplement: gkae1223_Supplemental_Files [file gkae1223_supplemental_files.zip › Supplemental Information - Rapid degradation of Histone Deacetylase 1 (HDAC1) reveals essential roles in both gene repression and active transcription - updating for NAR reviews.pdf]

Supplemental Information

Rapid degradation of Histone Deacetylase 1 (HDAC1) reveals essential roles in both gene repression and active transcription

David M English[1], Samuel N Lee[1], Khadija A Sabat[1], India M Baker[1,2], Trong Khoa Pham[3,4], Mark O Collins[3,4] and Shaun M Cowley[1,*]

[1] Department of Molecular and Cell Biology, Henry Wellcome Building, University of Leicester, Leicester, LE1 7RH, United Kingdom

[2] Cambridge Stem Cell Institute & Department of Haematology, Jeffrey Cheah Biomedical Centre, Cambridge Biomedical Campus, University of Cambridge, Puddicombe Way, Cambridge, CB2 0AW, United Kingdom

[3] School of Biosciences, University of Sheffield, Sheffield S10 2TN, UK

[4] biOMICS Mass Spectrometry Facility, University of Sheffield, Sheffield S10 2TN, UK

*To whom correspondence should be addressed

Smc57@le.ac.uk

Tel: +44 (0)116 2297098

Fig S1

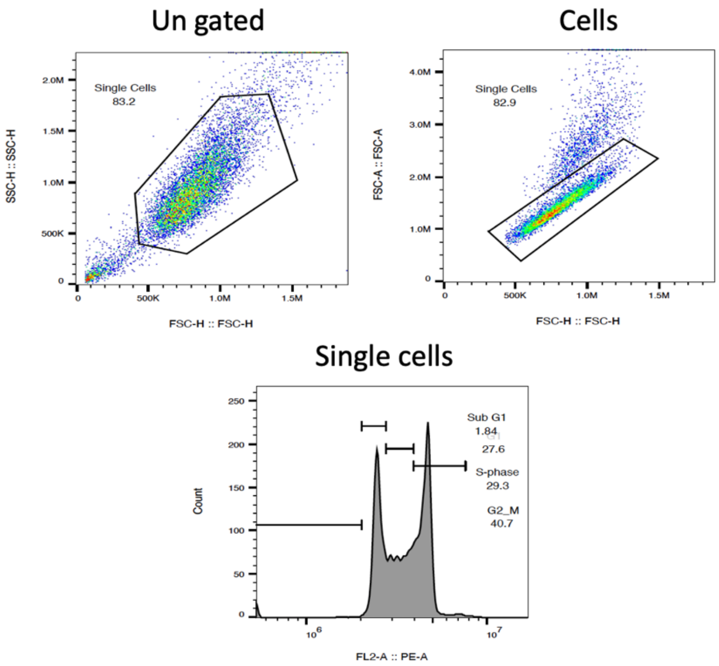

Figure S1. **Gating strategy used for PI FACS analysis.** A minimum of 10,000 events within the single cells gate were captured and analysed using FlowJo.

Fig S2

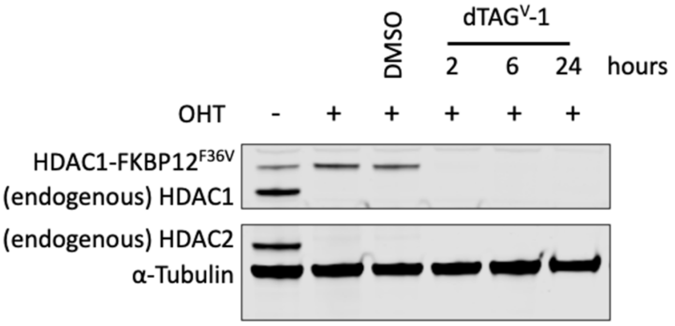

Figure S2. **dTAG^{V}-1 degrades HDAC1-FKBP12^{F36V} efficiently.** Western blot showing endogenous HDAC1 and HDAC1-FKBP12^{F36V} (detected with α-HDAC1), endogenous HDAC2 (detected with α-HDAC2) when treated with OHT and dTAG^{V}-1 as indicated, α-tubulin shown as a loading control.

Fig S3

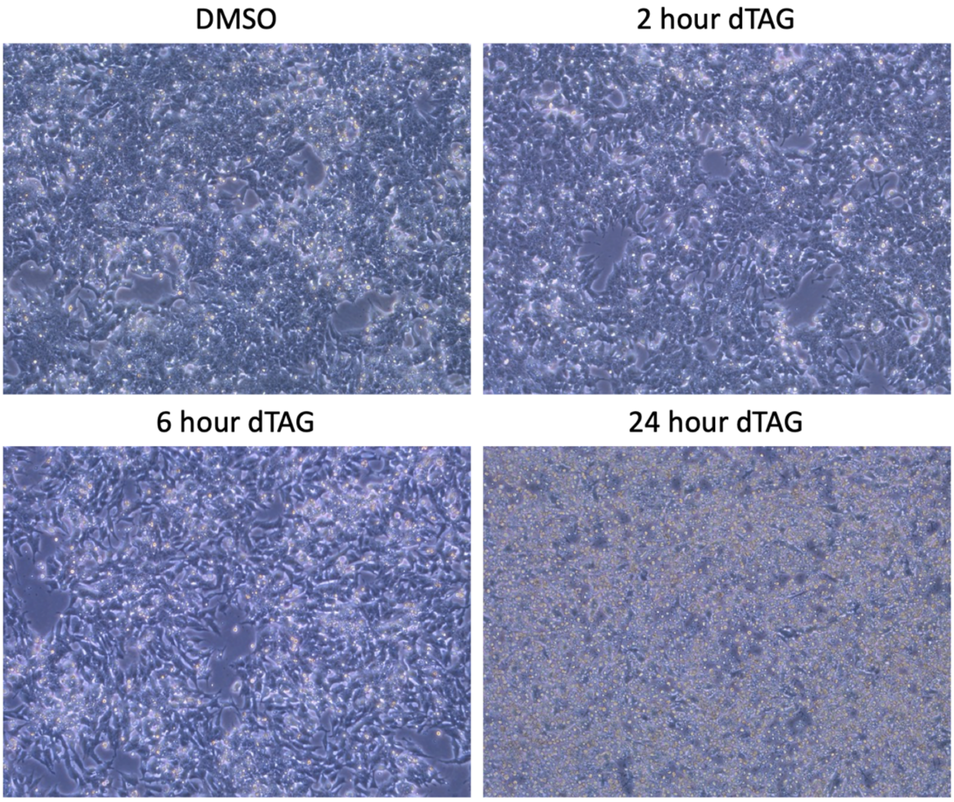

Figure S3. **HDAC1-FKBP12^{F36V} degradation causes cell death within 24 hours**. Images showing the effect of the indicated treatment time with 50 nM dTAG-13 on HDAC1-FKBP cells (images shown at 10x magnification).

Fig S4

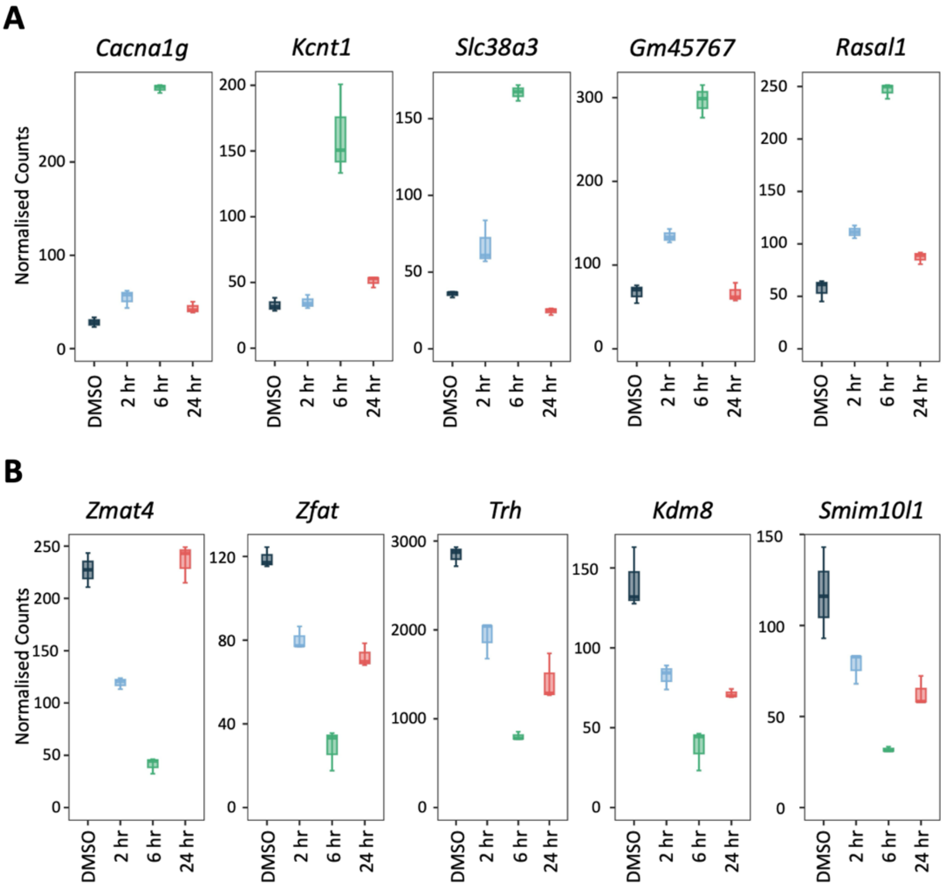

Figure S4. **A subset of genes is dysregulated only at 6 hours following HDAC1-FKBP12^{F36V} degradation.** (**A**) Boxplots showing the normalised count values for 5 genes which are only significantly upregulated (padj < 0.01, $\log_2$ fold change > 1) at 6 hours after HDAC1-FKBP12^{F36V} degradation. (**B**) Boxplots showing the normalised count values for 5 genes which are only significantly downregulated (padj < 0.01, $\log_2$ fold change > -1) at 6 hours after HDAC1-FKBP12^{F36V} degradation.

Fig S5

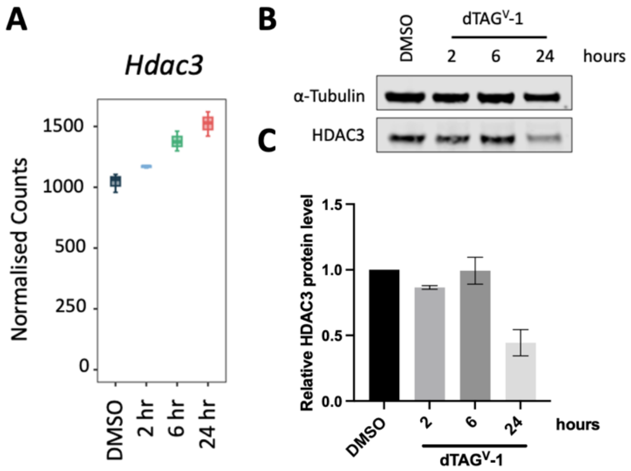

Figure S5**. There is not an increase in HDAC3 protein levels to compensate for loss of HDAC1/2**. (**A**) Boxplot showing normalised counts of *Hdac3* mRNA following the indicated dTAG^{V}-1 treatment times (n=3 biological replicates). (**B**) Western blot showing HDAC3 proteins levels with the indicated dTAG^{V}-1 treatment times, α-tubulin shown as a loading control. (**C**) Quantification of blot shown on (B), bars show the relative levels of HDAC3 protein normalised to α-tubulin (n=3 biological replicates, +/- SD).

Fig S6

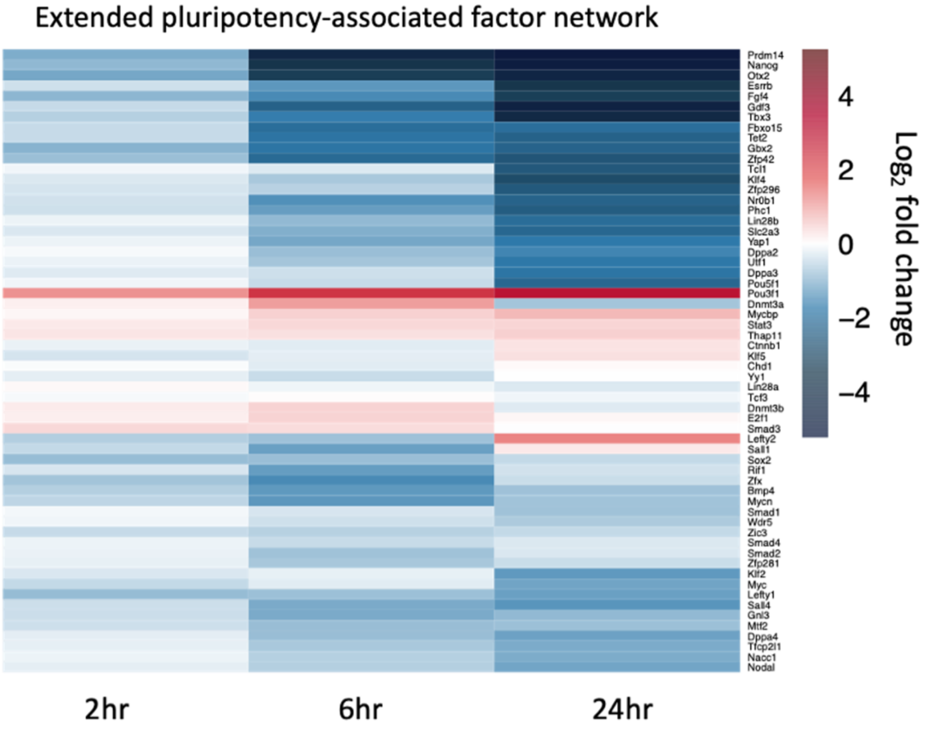

Figure S6. **HDAC1-FKBP12^{F36V} degradation causes a general downregulation of the extended pluripotency-associated factor network.** Heatmap indicating the log_2 fold change values for the indicated pluripotency-associated genes at the indicated time points following dTAG^{V}-1 treatment (n=3 biological replicates).

Fig S7

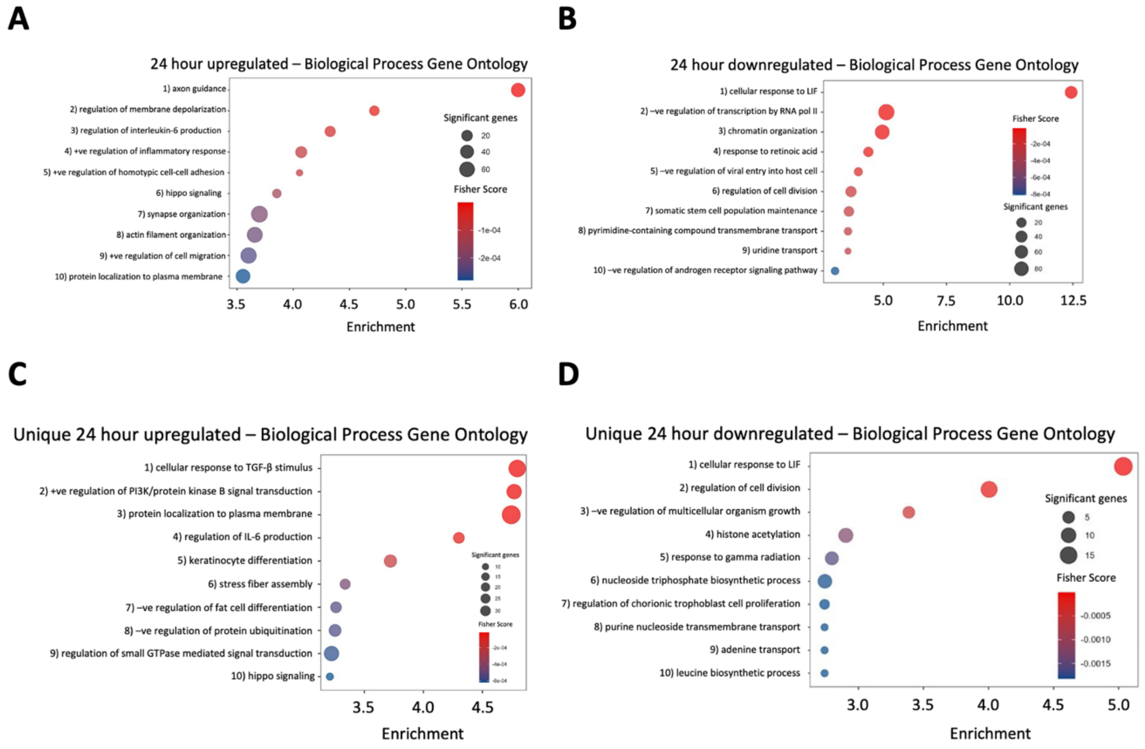

Figure S7. **24 hour GO terms reveal that a longer period of HDAC1-FKBP12^{F36V} degradation also affects key ESC processes.** (**A**, **B**) The 10 most enriched biological process gene ontology (GO) terms associated with the upregulated (A) or downregulated (B) genes (padj <0.01, log2 fold change > +1) following 24 hours of HDAC1-FKBP12^{F36V} degradation. (**C**, **D**) The 10 most enriched biological process gene ontology (GO) terms associated with the upregulated (A) or downregulated (B) genes that are only differentially expressed (padj <0.01, log2 fold change > +1) following 24 hours of HDAC1-FKBP12^{F36V} degradation.

Fig S8

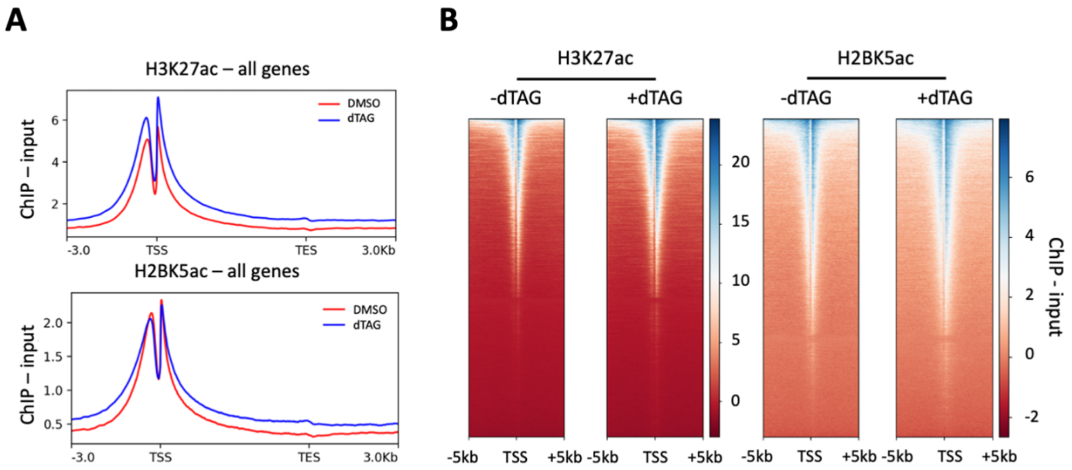

Figure S8. **H2BK5ac spreads due to HDAC1-FKBP12^{F36V} degradation.** (**A**) Metaplots showing the average signal (ChIP - Input) for H3K27ac and H2BK5ac across all genes following 6 hours of 100 nM dTAG^{V}-1 or DMSO treatment as indicated, including the regions -/+ 3 kb from the transcription start site (TSS) and transcription end site (TES) respectively (n=3 biological replicates). (**B**) Heatmaps showing the ChIP - Input values for H3K27ac and H2BK5ac following 6 hours of 100 nM dTAG^{V}-1 treatment as indicated, for the regions -/+ 5 kb from the TSS of all genes (n=3 biological replicates).

Fig S9

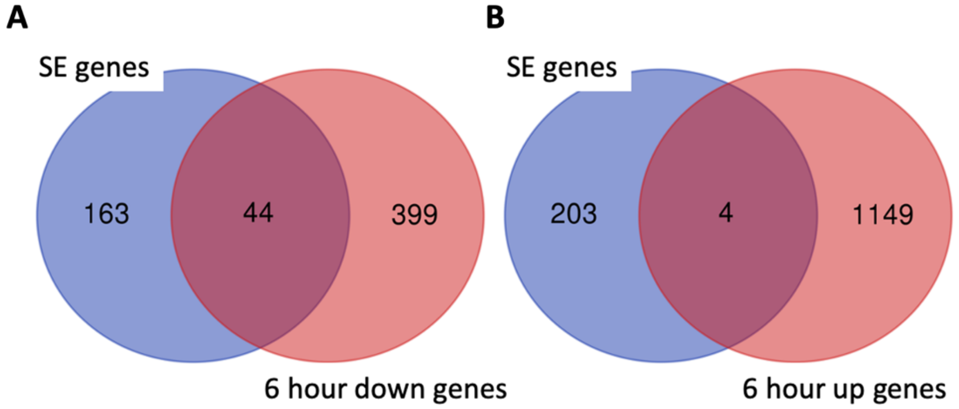

Figure S9. **Reduced super-enhancer (SE) acetylation is specifically linked to downregulation of gene expression.** (**A, B**) Venn diagrams showing the overlap between the SE regulated genes that we have assigned and the downregulated (A) or upregulated (B) genes determined by RNA-seq following 6 hours of dTAG^{V}-1 treatment.

Fig S10

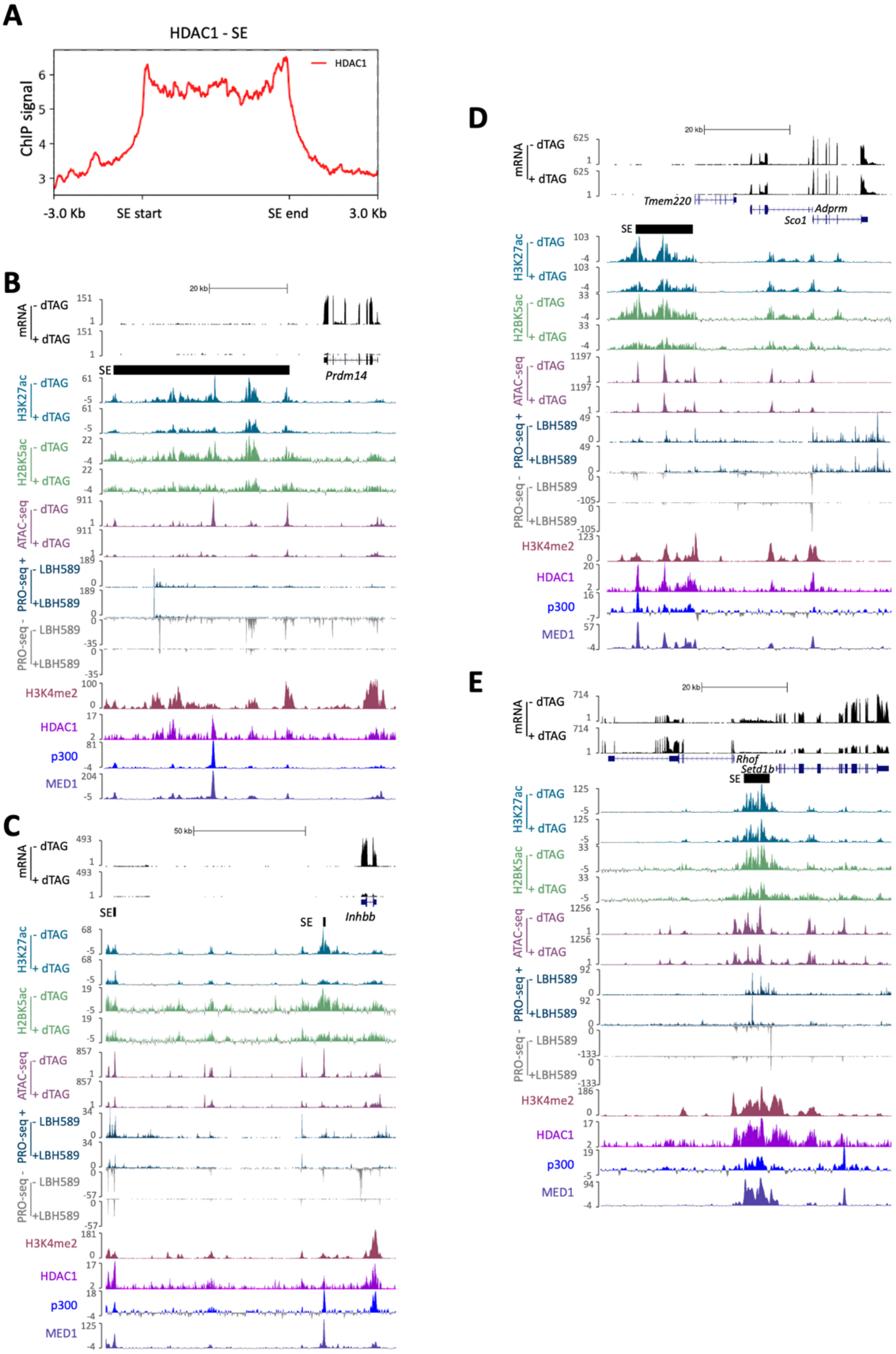

Figure S10. **SE accessibility is reduced at super-enhancers following HDAC1 degradation.** (**A**) Metaplot showing HDAC1 is bound to SE regions in ES cells using data from (53). (**B, C, D, E**) Tracks from the UCSC genome browser (54) showing the effect of 6 hours of 100 nM

dTAG^{V}-1 treatment on mRNA, H3K27ac, H2BK5ac and chromatin accessibility at the *Prdm14* (B) and *Inhbb* (C) loci. The SEs which were reassigned from Rhof to Setdb1 (D) and from Tmem220 to Sco1 (E). Additional tracks showing PRO-seq data indicating RNAP II recruitment following 6 hours of treatment with the pan HDAC inhibitor LBH589 (64), H3K4me2 (produced in our lab previously), HDAC1 binding (53) and p300/MED1 (51, 52) are also shown.
