# Supplementary material for: Rapid degradation of histone deacetylase 1 (HDAC1) reveals essential roles in both gene repression and active transcription
Source: Nucleic Acids Res. 2024 Dec 19;53(4):gkae1223. doi: 10.1093/nar/gkae1223 (PMC11879047; doi:10.1093/nar/gkae1223)
Supplement: gkae1223_Supplemental_Files [file gkae1223_supplemental_files.zip › Supplementary table legends .docx]

Supplementary Table S1. Table showing full breakdown of PI FACS data following 2, 6 or 24 hours of 100 nM dTAG^V^-1 treatment.

Supplementary Table S2. Table showing full breakdown acetylomic mass spectrometry data following 2 or 6 hours of 50 nM dTAG-13 treatment.

Supplementary Table S3. Table showing full breakdown of RNA-seq data displaying all genes and differentially expressed genes following 2, 6 or 24 hours of 100 nM dTAG^V^-1 treatment.

Supplementary Table S4. Table showing super-enhancer to gene designation and relative changes in H3K27ac and H2BK5ac at the promoters and super-enhancers of super-enhancer regulated genes.
